# Exosomes Derived From Alveolar Epithelial Cells Promote Alveolar Macrophage Activation Mediated by miR-92a-3p in Sepsis-Induced Acute Lung Injury

**DOI:** 10.3389/fcimb.2021.646546

**Published:** 2021-05-10

**Authors:** Fen Liu, Wei Peng, Jiaquan Chen, Zeyao Xu, Rong Jiang, Qiang Shao, Ning Zhao, Kejian Qian

**Affiliations:** Department of Critical Care Medicine, The First Affiliated Hospital of Nanchang University, Nanchang, China

**Keywords:** sepsis, acute lung injury, exosomes, miR-92a-3p, alveolar macrophage, alveolar epithelial cells

## Abstract

Acute lung injury (ALI) induced by sepsis is characterized by disruption of the epithelial barrier and activation of alveolar macrophages (AMs), which leads to uncontrolled pulmonary inflammation. However, effective treatments for ALI are unavailable. The exact mechanism by which the initial mediator of alveolar epithelial cells (AECs) induces inflammation remains elusive. Here we investigated the roles of AEC-derived exosomes in AM activation and sepsis-induced ALI *in vivo* and *in vitro*. Cecal ligation and puncture (CLP) was utilized to establish septic lung injury model in rats. The effect of exosomal inhibition by intratracheal GW4869 administration on lung injury was investigated. To assess the effects of AEC-derived exosomes on ALI, we treated the rat alveolar epithelial cell line RLE-6TN with LPS to induce cell damage. Exosomes from conditioned medium of LPS-treated AECs (LPS-Exos) were isolated by ultracentrifugation. The miRNAs in LPS-Exos were screened by miRNA expression profile analysis. The effects of miR-92a-3p on the function of AMs were studied. We found that intratracheal GW4869 administration ameliorated lung injury following CLP-induced ALI. LPS-Exos were taken up by AMs and activated these cells. Consistently, administration of LPS-Exos in rats significantly aggravated pulmonary inflammation and alveolar permeability. Moreover, miR-92a-3p was enriched in LPS-Exos and could be delivered to AMs. Inhibition of miR-92a-3p in AECs diminished the proinflammatory effects of LPS-Exos *in vivo* and *in vitro*. Mechanistically, miR-92a-3p activates AMs along with pulmonary inflammation. This process results in activation of the NF-κB pathway and downregulation of PTEN expression, which was confirmed by a luciferase reporter assay. In conclusion, AEC-derived exosomes activate AMs and induce pulmonary inflammation mediated by miR-92a-3p in ALI. The present findings revealed a previously unidentified role of exosomal miR-92a-3p in mediating the crosstalk between injured AEC and AMs. miR-92a-3p in AEC exosomes might represent a novel diagnostic biomarker for ALI, which may lead to a new therapeutic approach.

## Introduction

Sepsis, one of the most fatal diseases worldwide, is defined as life-threatening organ dysfunction, mainly due to dysregulated host responses to infection (Chen et al., 2011; Verdonk et al., 2017). The lung is the most vulnerable and critical organ that is injured during sepsis. More than half of patients with sepsis progress to acute lung injury (ALI) or acute respiratory distress syndrome (ARDS) (Sevransky et al., 2009). The mortality rate of ALI is greater than 40%, resulting in major economic and social burdens (Fein and Calalang-Colucci, 2000; Iscimen et al., 2008). ALI is characterized by an overwhelming and uncontrolled intrapulmonary inflammatory response that involves a number of activated inflammatory cells and cytokine release (Matthay et al., 2012). However, the pathogenesis associated with the development of ALI is still unclear, which might contribute to high mortality rates and the lack of effective treatments during ALI. Emerging evidence suggests that intercellular interactions play an important role in the regulation of lung injury progression, which is becoming a new research hotspot in the field of critical care medicine.

Alveolar macrophages (AMs), the main immune cells residing in the lung tissue, play a central role in the pathogenesis of ALI by releasing excessive inflammatory cytokines, and promoting neutrophil infiltration and tissue damage in the lung. AMs continuously receive exogenous signals from surrounding or other distant cells. Recent studies have found that AMs are closely related to lung structural cells in the lung (Misharin et al., 2013; Arora et al., 2018). Alveolar epithelial cells (AECs) are structural cells with a large surface area that form a barrier serving as the first line of pathogen defence in the alveolus. During the process of lung injury, alveolar epithelial cells are usually the initial site that is destroyed. Accumulating evidence has demonstrated that the alveolar epithelium is not only injured during this process but is also a driving force in the progression of lung diseases (Sunil et al., 2002; Sharma et al., 2007). When exposed to external pathogenic microorganisms, the alveolar epithelium is involved in the recruitment of various inflammatory mediators and immune cells including AMs. However, the mechanisms by which AEC-derived inflammatory signals are transmitted to macrophages during the development of septic lung injury remain largely unknown. Therefore, further clarification of the molecular mechanism of information transmission between alveolar epithelial cells and alveolar macrophages is urgently needed. This issue is important for the development of early biomarkers and the exploration of new therapeutic targets for septic lung injury.

Recent evidence shows that exosomes play a key role in the transmission of signals between cells (Mathieu et al., 2019). Almost all types of cells have been shown to secrete exosomes, which can be detected in biological fluids, including BALF, urine, and breast milk. Exosomes contain a variety of biologically active substances including proteins, nucleic acids, miRNAs, and lipids (Wozniak et al., 2020). The components in exosomes will change according to different environments. In recent years, studies emerging documents have focused on miRNAs in exosomes (Valadi et al., 2007; Squadrito et al., 2014). miRNAs are short noncoding RNAs with a length of approximately 21-23 nucleotides. miRNAs regulate the expression of target genes by inhibiting translation or inducing RNA degradation (Lee et al., 2009). miRNAs play an important role in the post-transcriptional regulation of biological processes. A variety of miRNAs have been identified in exosomes. The released miRNAs can bind to the 3’-UTR of the target gene mRNA. Numerous studies have found that exosomes play an important role in the development of acute lung injury mediated by miRNAs (Jiang et al., 2019). For septic lung injury, studies have found that BALF contains a large number of exosomes encapsulating miRNAs in an LPS-induced ALI model. Exosomes in BALF can induce inflammation in recipient cells (Soni et al., 2016; Yuan et al., 2018). However, it is unknown whether exosomes derived from AECs regulate AMs inflammation through encapsulated miRNAs. In the present study, we proved that exosomal miRNAs released by AECs could initiate intrapulmonary inflammation by conveying inflammatory signals to activated macrophages.

## Materials and Methods 

### Reagents and Antibodies

LPS, GW4869 and PKH-67 were purchased from Sigma-Aldrich. The following antibodies were used: anti-CD68, anti-CD9, anti-CD63, anti-PTEN, anti-Alix, anti-Akt, anti-p-Akt, goat Anti-Rabbit IgG H&L (Alexa Fluor® 555) (Abcam), anti-p65, and anti-p-p65(Beyotime); miR-92a-3p mimic, mir-92a-3p inhibitor, control RNAs and the primers for miRNAs were all purchased from Guangzhou RiboBio.

### Cell Culture

The rat alveolar macrophage cell line NR8383 was purchased from the Chinese Academy of Sciences Cell Bank. Cells were cultured in HamF-12K culture medium (Wisent, Canada) containing 15% foetal bovine serum (Gibco, USA) at 37°C in a 5% CO_2_ incubator. The culture medium was changed once every 2 to 3 days and cell passaging was performed once every 3 to 4 days. The rat alveolar epithelial cell line RLE-6TN was also purchased from the Chinese Academy of Sciences Cell Bank. The cells were routinely maintained in DMEM culture medium (Boster, China) containing 10% foetal bovine serum, 100 IU per ml penicillin and 100 μg per ml streptomycin at 37°C with 5% CO_2_ in an incubator. For *in vitro* experiments, AECs were cultured in the presence or absence of 1 µg/ml LPS in serum-free DMEM for 24 h, and the supernatant was collected for exosome isolation.

### Construction of the Rat Model of Sepsis-Induced Acute Lung Injury

Male SD rats weighing approximately 100g (4-5 weeks old) were purchased from Animal Experimental Research Center of Nanchang University, China (Certificate of Conformity: SYXK2015-0001). The rats were fed SPF-level maintenance food from Jiangsu Xie Tong Corporation (Nanjing) and had free access to drinking water. Housing conditions included a 12 h light and dark cycle, temperature of 21–22°C and cage replacement once/week. Animal experimental procedures and protocols were approved by the ethics committee of the Animal Experimental Research Center of Nanchang University (No. NCDXSYDWLL-2017619).

To construct the model of septic lung injury, we used cecal ligation and puncture (CLP) for sepsis, which has been previously described by Rittirsch et al. (2009). Briefly, a median incision of 1-2cm was made, and the caecum was exteriorized and ligated distal to the ileocecal valve (without causing intestinal obstruction). The caecum was perforated eight times using a 20-gauge needle and squeezed gently to extrude a small amount of faecal contents from the punctured caecum into the abdominal cavity. The caecum was then returned to the peritoneal cavity and the incision was closed using two layers of sutures.

### Exosomal Isolation

The cell culture medium (CCM) from AECs was harvested after exposure to LPS for 24 h of incubation and centrifuged at 300 × g for 10 min, 2000 × g for 20 min, and 10 000 × g for 30 min at 4°C. The supernatant was subsequently collected and filtered through 0.22 μm filters (Millipore, MA, USA) and later centrifuged in an ultracentrifuge Beckman Coulter Optima TM L‐80XP at 100 000 × g, for 70 min at 4°C to pellet exosomes. Next, the exosome pellets were washed with sterile PBS and subjected to another cycle of ultracentrifugation at 100 000 × g for 70 min at 4°C. Finally, the pelleted exosomes were carefully reconstituted in sterile PBS. This was LPS-treated AEC exosomes (LPS-Exos). The exosomes extracted from the normal AEC culture supernatant were used as a control (Ctrl-Exos).

### Exosome Characterization and Inhibition of Exosome Release

For the transmission electron microscopy (TEM) analysis of morphology 20 μL exosome suspensions were dropped on a sample-loaded copper mesh with a pore size of 2 nm. Samples were negatively stained with 3% phosphotungstic acid, pH 7.5, and observe the shape of the exosomes was observed through transmission electron microscopy. The diameters of the exosomes were quantified by nanoparticle tracking analysis (NTA) with a NanoSight LM10 instrument (Nano Sight Limited, Amesbury, UK) as described. Western blot analysis was performed to evaluate the protein expression of the exosome markers CD63 and CD9 by using specific antibodies. To inhibit the intrapulmonary release of exosomes in septic lung injury, we instilled GW4869 into the airway at a dose of 2.5 μg/g before cecal ligation and puncture.

### Exosomal miRNA Microarray Assay

A miRNA microarray was used to investigate differentially expressed miRNAs between LPS-Exos and Ctrl-Exos. Briefly, total RNA was extracted and purified using the miRNeasy Mini Kit (Qiagen). Exosomal miRNA microarray hybridization, data generation, and normalization were performed by Shanghai Biotechnology Corporation (Shanghai, China), using the Agilent Rat miRNA Microarray (8×15K, version 21.0; Agilent Technologies, USA). Microarray slides were sequenced on an Agilent Microarray Scanner, and the raw data were normalized by the quantile algorithm GeneSpring Software 12.6.1 (Agilent Technologies). To comprehensively analyse the microarray data, we applied filtering criteria (≥2-fold change, P < 0.05). After miRNA microarray, cDNA was generated *via* the miRNA cDNA synthesis kit (Thermo, USA) according to the manufacturer’s protocol. Thereafter, real-time PCR was carried out using cDNA samples with the miRNA qPCR Assay Kit (TaKaRa, Japan) and the 2-ΔΔCt method was used to calculate the relative change in miRNA expression after normalization to levels of U6 small nuclear RNA.

### Exosomal Labelling and Confocal Microscope

The fluorescent dye PKH-67 (400 μL) was added to the exosome suspension, incubated at room temperature for 5 min, blocked with an equal volume of exosome-free serum, and washed twice with PBS to remove unbound dye. PKH-67-labelled exosomes were added to culture alveolar macrophages (NR8383), After incubation for 12 h, NR8383 cells were fixed, washed, and viewed with a confocal laser scanning microscope (Olympus).

### Transfection

Alveolar epithelial cells were transfected with mir-92a-3p mimic (100 nM), mir-92a-3p inhibitor (4 μM) and control RNAs using Lipofectamine 2000 according to the manufacturer’s instructions.

### Plasmid Construction and Luciferase Assay

The complete rat PTEN3’-UTR segment was amplified by PCR using genomic DNA as a template. The PCR product was subcloned into the psiCHECK-2 vector following the manufacturer’s protocol (Promega) to obtain wild-type psi-CHECK-PTEN-3’UTR (wt). Mutations (Mut-UTRs) were generated by a site-directed mutagenesis kit (Stratagene; Agilent Technologies, Inc., Santa Clara, CA, USA), yielding the Mut-UTR plasmid. A mutant of potential miR-92a-3p binding sites in WT-3′-UTR with a mutation (mut) of complementary sequences (termed psi-CHECK-2-PTEN-mut-3′-UTR) was generated using the QuikChange II Site-Directed Mutagenesis Kit (Stratagene, USA). Plasmid DNA was sequenced to ensure its authenticity. For the luciferase assay, 293T cells were cultured in a 96-well plate, and cells in each well were transfected with 0.2 mg luciferase reporter constructs described above at the same time with miR-92a-3p mimic (100 nM) or control RNAs using Lipofectamine 2000 (Invitrogen). After 48 h, cells were assayed using luciferase assay kits (Promega).

### The Ratio of Dry to Wet Lung Tissue

The upper lobe of the right lung was wiped with dry sterile gauze and then weighed (W). Later it was placed in an oven at a constant temperature of 60°C to dry for 72 h and weighed again (D) to calculate the dry-wet weight ratio of lung tissue (W-D)/W.

### Evans Blue Content

For evaluation of pulmonary vascular permeability, each group of rats was injected with 1% Evans Blue 2 ml/kg *via* the tail vein. The mice were sacrificed 0.5 h after the injection, and the left lung tissue (~100 mg) was taken, cut into pieces, and roasted at 50°C for 72 h until a constant weight was achieved. Another 100 mg of right lung tissue was taken, and formamide was added to wet lungs at 1 ml/100 mg, homogenized, and placed in a 50°C water-isolated incubator for 24 h. After centrifugation, the supernatant was collected, the Evans blue content was measured by a spectrophotometer, and the average value of both sides was used.

### Total Protein Content in BALF and Plasma

Total protein content in BALF and plasma was detected by a Bradford protein concentration quantitation kit (Solarbio Lifesciences, China).

### Histopathology

The right lower lobe lungs of the mice were soaked in formalin for 24 h and then sent to the tissue embryo laboratory, where each lung sample was sliced into 4-μm thick sections. For analysis of tissue inflammation, the lung sample sections were subsequently stained with haematoxylin and eosin. Lung injury scores were estimated by the Smith’s scoring method, with a higher score indicating more severe injury.

### Immunohistochemistry

After deparaffinization and hydration, the lung tissue sections (4 μm) were incubated in 3% H_2_O_2_ for 10 min to block endogenous peroxidase activity for 10 min. The sections were washed three times with PBS and incubated in normal goat serum for 15 min to block nonspecific binding. They were stained with anti-PTEN and anti-CD68 antibodies overnight at 4°C and then incubated with a goat anti-rabbit IgG or donkey anti-goat IgG antibody for 1 h at room temperature. Peroxidase activity was visualized using a diaminobenzidine tetrahydrochloride (DAB) solution (Cell Signaling Technology, USA). The sections were evaluated with an optical microscope (Olympus Optical, Tokyo, Japan).

### Cytokine Enzyme-Linked Immunosorbent Assay (ELISA)

The levels of TNF-α, IL-1β and IL-6 were determined by ELISA kits following the manufacturer’s instructions. Standard curves were plotted to calculate concentrations of the inflammatory factors in samples.

### Western Blot

Cells or lung tissue homogenate was lysed using RIPA lysis buffer (Beyotime Biotechnology, Shanghai) containing a protease inhibitor cocktail for 30 min. Total protein was quantified by a BCA kit (Beyotime Biotechnology, China), and denatured by boiling in water. Protein suspension was loaded in SDS-PAGE gels. Proteins were separated and electrophoretically transferred onto a polyvinylidene fluoride membrane using an electroblotting apparatus (Bio-Rad, USA). The membrane was blocked in 5% bovine serum albumin for 60 min at room temperature, followed by incubation with antibody diluent for overnight at 4°C. After incubation with secondary HRP-conjugated antibody for 1 h at room temperature, the protein signals on the membrane were detected using a gel imaging scanning system (Bio-Rad, USA), and quantified by ImageJ (National Institutes of Health, USA) using β-actin as a reference protein.

### Real-Time Quantitative PCR

The total RNA of cells was isolated using RNA extraction kit (Omega, USA) and assayed by a NanoDrop system. A 20 μL reaction system was prepared according to the instructions of the reverse transcription reaction kit: 1) 2 μL of 5×gDNA Eraser Buffer, 1 μL of gDNA Eraser, 200 ng of total RNA, dd H_2_O to a total volume of 10μL, reacting at 42 °C for 2 min. 2) 10 μL of the reaction solution: 1 μL of Prime Script RT Enzyme Mix I, 1 μL of RT Prime Mix and 4 μL of 5 × Prime Script Buffer 2 and 4 μL of ddH_2_O. The reaction conditions were 37°C for 15 min → 85°C for 5 s → 4°C. RT-qPCR reaction system: 10 μL of SYBR^®^ Premix Ex Taq II (2×), 0.8μL of PCR Forward Primer (10 μM), 0.8 μL of PCR Reverse Primer (10 μM) and 0.4 μL of ROX Reference Dye (50×), 200 ng of RT reaction solution (cDNA solution), addition of ddH2O to a total volume of 20μL. Reaction conditions: 95°C for 30 s; followed by 40 cycles at 95°C for 5 s, 60° C for 30 s. After reaction completion, the amplification curve and the dissolution curve were confirmed. The mRNA levels were normalized against the mRNA level of β-actin and are represented as a fold change. The primers used are listed in **Table 1**.

**Table 1:** The sequences of primers for RT-PCR.

Primer name	Sequence (5’-3’)
IL-6-forward	GCTCTGGTCTTCTGGAGTTCC
IL-6- reverse	GAGTTGGATGGTCTTGGTCCT
IL-1β- forward	ACAAGGAGAGACAAGCAACGA
IL-1β- reverse	TCTGCTTGAGAGGTGCTGATG
TNF-α- forward	GGCAGCCTTGTCCCTTGAAGAG
TNF-α- reverse	GTAGCCCACGTCGTAGCAAACC
PTEN-forward	TGGATTCGACTTAGACTTGACC
PTEN-reverse	AGGATATTGTGCAACTCTGCAA
β-actin- forward	TACTGCCCTGGCTCCTAGCA
β-actin- reverse	TGGACAGTGAGGCCAGGATAG

IL-6, Interleukin-6; IL-1β, Interleukin-1β; TNF-α, Tumor necrosis factor-alpha.

### Statistical Analysis

The measurement data are presented as the mean ± standard deviation (SD). Multiple samples were compared by one-way analysis of variance to determine whether there were significant differences. The comparison of each group was performed by LSD-t test, and the Dunnett-t test was used to compare the ELISA results. P < 0.05 was considered statistically significant. All data were analysed by SPSS 19.0 software (IBM Corp. USA).

## Results

### Exosomes Participate in the Regulation of Inflammation in Sepsis-Induced Lung Injury

**Figure 1:**
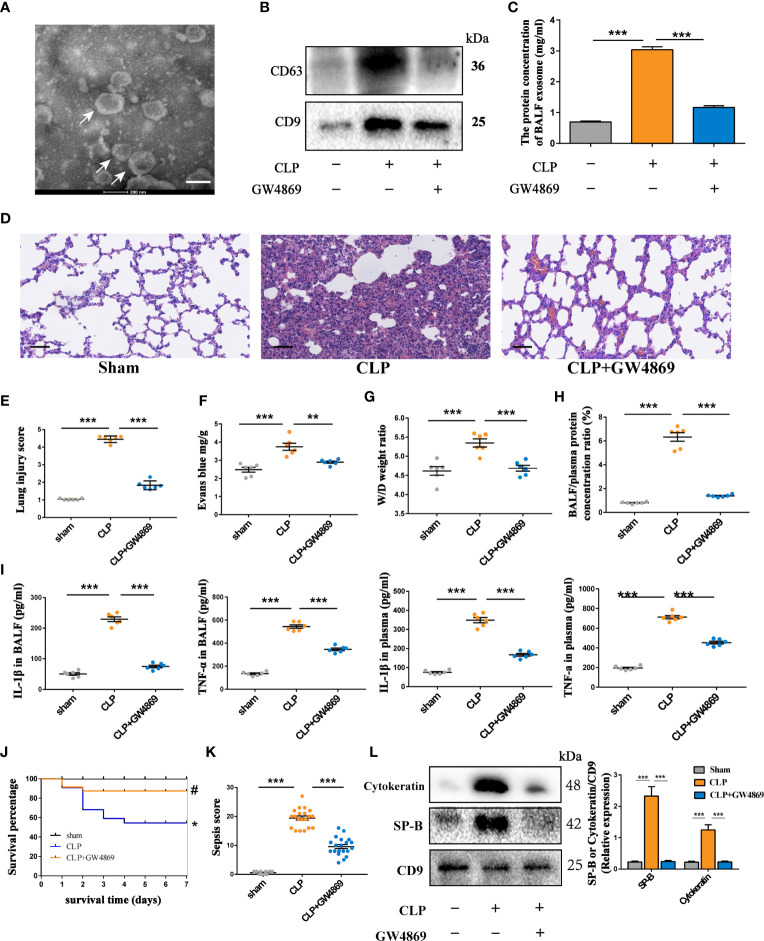
Inhibiting the secretion of intrapulmonary exosomes alleviates septic lung injury. Rats were treated with GW4869 (2.5 μg/g) through airway instillation. CLP procedures were conducted one hour post-treatment, and sham operated rats were used as negative controls. **(A)** Representative electron micrograph of exosomes purified from the BALF (indicated by white arrows). Scale bars, 200 nm. **(B)** Western blot assessment of exosomes showing the presence of CD63 and CD9 in BALF-derived exosomes. **(C)** Quantification of exosomal protein concentration in the BALF of each group of rats. **(D)** Histopathological examination of lung tissue by HE staining, Scale bars,50 µm. **(E)** Scoring the pathology of lung tissue damage using the Smith scoring method. **(F–H)** Changes in lung tissue oedema of rats under different treatments. **(F)** Evans blue content in lung tissue. **(G)** The wet/dry weight ratio in lung tissue. **(H)** The ratio of protein concentration in BALF to plasma. **(I)** The expression levels of IL-1β and TNF-α in BALF and plasma. **(J, K)** Survival rate of rats in each group within 7 days and the changes in sepsis score of each group. The Kaplan-Meier method combined with the log-rank test was used to compare multiple populations. Compared with the sham group, *P<0.05; compared with the CLP group, ^#^P<0.05; n=10 for the sham group, n=20 for the other groups. **(L)** Western blot assessment of the expression of epithelial cell-specific marker proteins SP-B and cytokeratin in exosomes derived from BALF and quantitative analysis of the protein grey value. Data are presented as the mean ± S.E.M. **P<0.001, ***P<0.001 compared between two groups. n=6 per group.

Exosomes are an important mediator of inflammatory information communication, but the role of exosomes in septic lung injury is poorly understood. Here, we explored the effects of exosomes on the progression of septic lung injury by inhibiting exosome release. Rats were subjected to cecal ligation and puncture (CLP) to generate a septic lung injury model. Twenty-four hours after CLP treatment, typical histological injuries in the lung, such as lung inflammation, an increased lung W/D weight ratio and an increased protein concentration in BALF, were observed (**Figures 1E–G**). These data suggest that a septic lung injury model in rats was successfully established. Exosomes isolated from the BALF of the rats with septic lung injury were morphologically characterized using transmission electron microscopy. As shown in **Figure 1A**, the isolated microvesicles displayed a round, cup-shaped morphology and were approximately 100 nm in diameter. Furthermore, upregulation of the expression of the exosome-specific markers CD63 and CD9 was detected by western blots in samples from the rats with septic lung injury (**Figure 1B**). To examine the function of exosomes in septic lung injury, we used GW4869, an exosome release inhibitor, to reduce exosome secretion in the lung. GW4869 treatment significantly decreased the protein concentration of BALF exosomes in the rats with septic lung injury (**Figures 1B, C**). The inflammatory cell infiltration in lung interstitial tissue, pulmonary capillary permeability, lung wet/dry ratio and levels of the IL-1β and TNF-α proteins in BALF were significantly reduced in the GW4869-treated rats compared to the vehicle-treated animals (**Figures 1D–I**). GW4869 treatment increased the survival rate of the septic rats and decreased the sepsis score in the lung (**Figures 1J, K**). These data suggested that exosomes derived from pulmonary cells act as detrimental factors during septic lung injury. Inhibiting the release of exosomes with GW4869 treatment protected the lungs from sepsis-induced damage.

The important roles of secreted extracellular vesicles derived from alveolar epithelial cells were documented in the regulation of lung injury (Bissonnette et al., 2020). However, little is known about the effects of alveolar epithelial cell-derived exosomes in sepsis-induced ALI. Interestingly, the expression of the AEC markers SP-B and cytokeratin was robustly upregulated in BALF exosomes in septic lungs. GW4869 treatment significantly reduced the content of exosomes derived from alveolar epithelium (**Figure 1L**). Taken together, these findings indicate that large amounts of AEC-derived exosomes might be a harmful factor in lung injury during sepsis.

### Exosomes Derived From LPS-Treated AECs Induced Acute Lung Injury *In Vivo*

**Figure 2:**
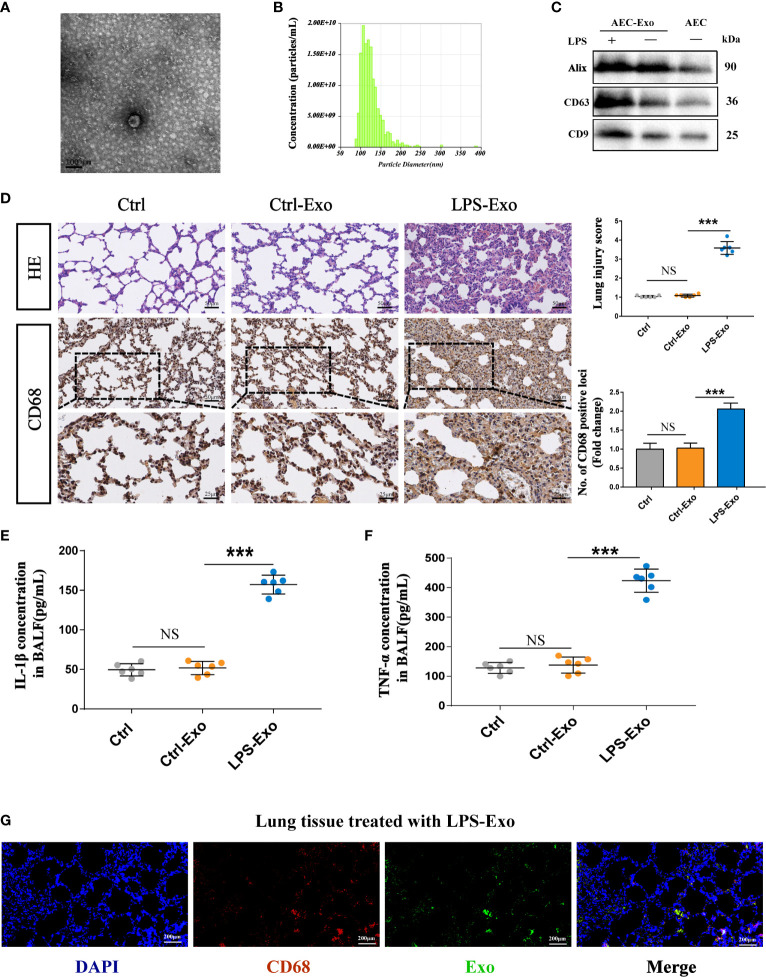
Exosomes derived from LPS-treated AECs induce acute lung injury. **(A–C)** Characterization of exosomes isolated from the supernatant of cultured AECs. **(A)** Electron micrograph of exosomes. Scale bar, 100 nm. **(B)** Exosome size distribution was measured by Nano Sight tracking analysis. **(C)** Alix, CD63 and CD9 protein expression in exosomes was quantified by western blot loaded with equal amounts of protein (40 μg). **(D–G)** Exosomes (2 mg/kg) were intratracheally transferred to SD rats through intratracheal, and analysis was performed 24 h after treatment (n = 6 for each group). **(D)** Lung histological changes and lung injury scores were assessed with HE staining. Immunohistochemical detection of CD68 positive macrophage infiltration in the lung. The numbers of CD68 positive loci were quantified with ImageJ software. Scale bar, 50 μm and 25 μm. **(E, F)** ELISAs showed the expression of proinflammatory cytokines (IL-1β and TNF-α) in the BALF. **(G)** LPS-Exos labelled with PKH-67 fluorescent dye were administered to rats. Immunofluorescence images showing the colocalization between exosomes (green) and CD68- labelled AMs (red) in the lung. Nuclei were counterstained with DAPI (blue). Data are presented as the mean ± S.E.M. ***P < 0.001 compared between two groups. NS, no significant difference. n=6 per group.

To further investigate the effects of AEC-derived exosomes on lung injury during sepsis, we used an alveolar epithelial cell line (RLE-6TN) as an *in vitro* model. LPS, a common stimulus during sepsis, was applied to stressed alveolar epithelial cells to mimic the inflammatory pathogenesis of septic lung injury. Exosomes were isolated from the supernatant of LPS-stressed AECs by ultracentrifugation. AEC-derived exosomes under LPS treatment (LPS-Exos) were cup-shaped vesicles, round or oval with clear margins, as shown by TEM (**Figure 2A**). The exosomes had a mean diameter of 107 nm with sizes ranging from 90 to 160 nm by nanoparticle tracking analysis (**Figure 2B**). Expression of exosome surface markers Alix, CD63 and CD9 was positive in isolated exosomes (**Figure 2C**). These results indicated that the extract from damaged AECs was similar to exosomes in size and shape.

To evaluate the effects of LPS-Exos on intrapulmonary inflammation and lung injury, we intratracheally administered either Ctrl-Exo or LPS-Exos (2.5 mg/kg, 100 μL) to the rats. Rats treated with PBS (100 μL) were used as negative controls. PBS and Ctrl-Exo treatment did not negatively affect lungs with intact alveolar cavities or rare inflammatory cells. However, LPS-Exo treatment substantially increased inflammatory cell infiltration in both alveolar and interstitial spaces with increasing thickness of the alveolar walls. LPS-Exo treatment significantly increased the lung injury score (**Figure 2D**). As shown in **Figures 2E, F**, LPS-Exos induced lung inflammation, as evidenced by increased cytokines including interleukin (IL-1β) and tumour necrosis factor (TNF)-α, compared to the Ctrl and Ctrl-Exos.

In parallel, fluorescence staining and immunohistochemistry were used in rat tissue to further examine the interaction between AEC exosomes and alveolar macrophages (AMs) (**Figure 2G**). Many CD68 positive macrophages were detected in the lungs after intratracheal LPS-Exos administration. Moreover, colocalization of PKH-67-labelled LPS-Exos and alveolar macrophages was easily observed. These results indicate that LPS-Exos could be taken up by AMs and that exosomes mediate the crosstalk between AECs and AMs. Most likely, AMs serve as target cells of LPS-Exos to exert inflammatory effects during lung injury.

### Exosomes Derived From LPS-Treated AECs Promote Alveolar Macrophage Activation *In Vitro*

**Figure 3:**
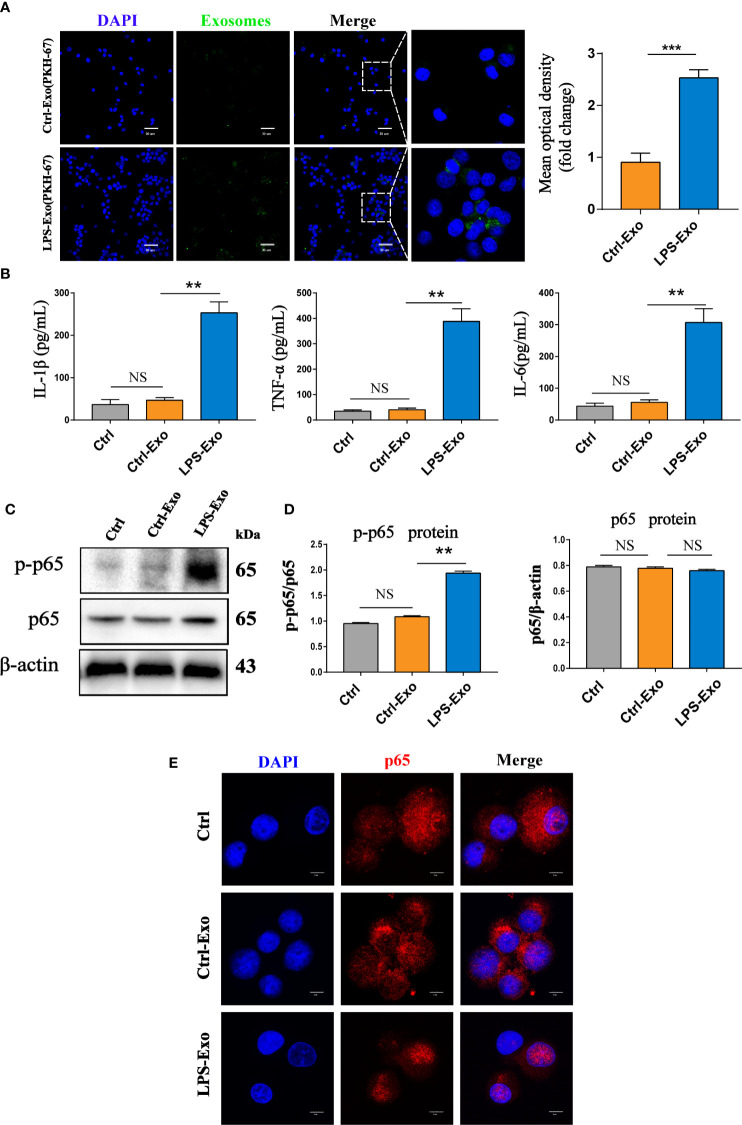
Internalized LPS-treated AECs promote AM activation. **(A)** PKH-67 labelled AEC exosomes were cocultured with recipient AMs for 12 h. Relative fluorescence intensity was quantified to identify the internalization of exosomes. Scale bar, 30μm. **(B)** Proinflammatory cytokine IL-1β, TNF-α and IL-6 levels in the supernatant of the AMs treated with AEC exosomes for 12 h. **(C, D)** Representative western blot and quantitative analysis of p-p65 and p65 in recipient AMs treated with AEC-derived exosomes with or without LPS stimulation. **(E)** Representative images of the nuclear translocation of p65, as observed by immunofluorescence assays. Immunofluorescence staining of p65 (red) and nuclei (blue) at 12 h following AMs were treated with LPS-Exos or Ctrl-Exos. Scale bar, 5 μm. Data are presented as the mean ± S.E.M. **P<0.01, ***P<0.001 compared between two groups. NS, no significant difference. n=3 per group.

Alveolar macrophages serve as the front line of defence against the invasion of pathogens during sepsis. The excessive secretion of inflammatory cytokines by activated alveolar macrophages is the key pathogenic factor of acute lung injury. To understand the effects of AEC-derived exosomes on alveolar macrophage activation, we stimulated alveolar macrophages with LPS-Exos for 12 h. To assess the internalization of exosomes, we cocultured PKH-67- labelled exosomes with DAPI- labelled AMs for 12 h. As shown in **Figure 3A**, AMs took up fluorescently labelled LPS-Exos. The levels of proinflammatory cytokines including TNF-α, IL-6 and IL-1β were substantially increased in the LPS-Exo-exposed macrophages compared to those in the Ctrl and Ctrl-Exo groups (**Figure 3B**). This change was also accompanied by increased expression of p-p65 protein (**Figures 3C, D**) and enhanced p65 translocation from the cytoplasm to the nucleus (**Figure 3E** and **Figure S1** in the **Supplemental Material**). These data suggested that exosomes derived from LPS-treated AECs induce activation of the NF-κB signalling pathway in AMs. Exosomes derived from normal AECs had no effects on the release of inflammatory factors or the activation of the inflammatory signalling pathway in AMs. Taken together, these results indicated that LPS-Exos could be efficiently taken up by alveolar macrophages to induce inflammation, which suggests a potential role of LPS-Exos in activating AMs to release inflammatory factors.

### miRNA Profile of AEC Exosomes in LPS-Induced Alveolar Epithelial Inflammation

**Figure 4:**
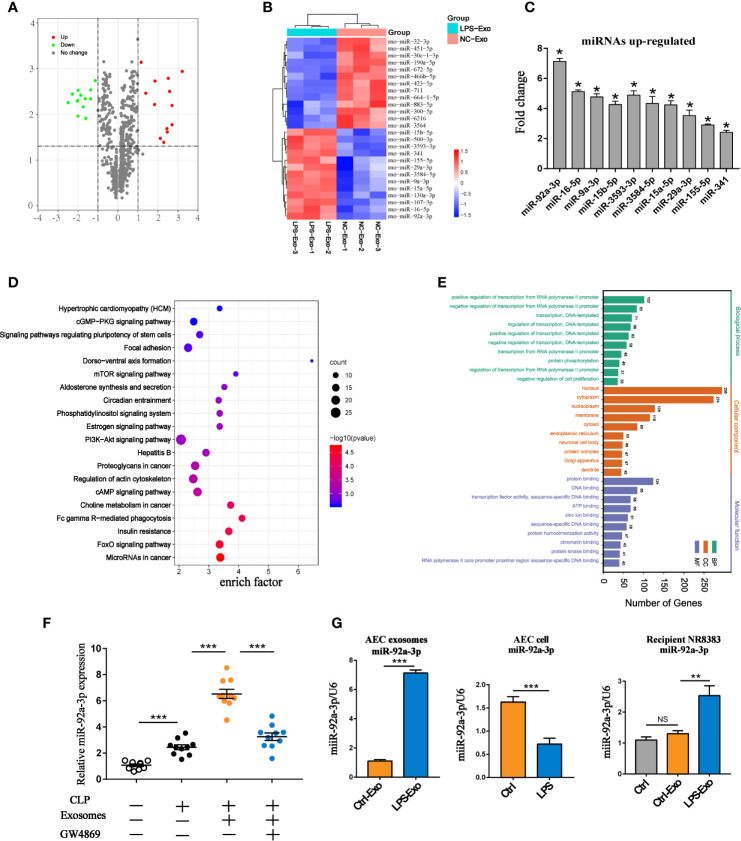
miRNA expression profile of LPS-treated AEC exosomes. **(A)** The volcano plot of differentially expressed miRNAs between the LPS-Exo and AEC-Exo groups (fold changes ≥2 and P<0.05), compared with the Ctrl-Exo group. The miRNAs with upregulated expression are represented by red dots, those with downregulated expression arerepresented by green dots and those with non-significantly changed expression are shown as grey dots. The vertical lines correspond to upregulation or downregulation by 2-fold, and the horizontal line represents the P value of 0.05. **(B)** The heatmap shows the differentially expressed miRNAs. **(C)** Validation of the expression levels of the top 10 upregulated miRNAs in LPS-Exos. Ctrl-Exos were used as a negative control. **(D, E)** Kyoto Encyclopedia of Genes and Genomes (KEGG) analysis and Gene Ontology (GO) were performed on the predicted target genes of miR-92a-3p. Ctrl-Exos and LPS-Exos represent exosomes isolated from the control and LPS-treated AECs, respectively. **(F)** The expression of miR-92a-3p in BALF or BALF-derived exosomes isolated from the septic rats. Rats were treated with GW4869 (2.5 µg/g) through airway instillation. An hour post-treatment, CLP procedures were conducted. Sham operated rats were used as negative controls. n=10 for each group. **(G)** Expression of miR-92a-3p in AEC derived exosomes, AECs and recipient AMs treated with AEC derived exosomes. Data are presented as the mean ± S.E.M. *P<0.05, **P<0.01, ***P<0.001 compared Brtween two groups. NS, no significance. n=3 per group.

miRNAs encapsulated in exosomes are believed to be important mediators of information exchange between cells. To further identify the key miRNAs in LPS-Exo-treated alveolar macrophages, we used a miRNA microarray to analyse the differences in their miRNA expression profiles between the LPS-Exo and Ctrl-Exo groups. Exosomes derived from normal alveolar epithelial cells were used as controls. A total of 760 miRNAs were detected in LPS-Exo- and Ctrl-Exo-exposed samples. Among them, 26 miRNAs were found to be differentially expressed between these two exosomes (minimum fold change of two and adjusted p-value < 0.05), of which 13 were upregulated and 13 were downregulated (**Figures 4A, B**). The expression levels of the top 10 upregulated miRNAs were validated by qRT-PCR (**Figure 4C**). The expression level of miR-92a-3p showed the greatest increase, and this molecule was previously reported to regulate inflammation (Kuo et al., 2019). As shown in **Figures 4D, E**, the TargetScan database was applied to predict the target genes of miR-92a-3p. The predicted target genes of miR-92a-3p were significantly enriched in immune- and inflammation-related pathways, such as the PI3K-Akt, FOXO and mTOR signalling pathways, as shown by KEGG and GO enrichment analyses. The expression of miR-92a-3p in septic BALF was examined, showing that miR-92a-3p expression was significantly increased in the BALF of the septic rats compared to that of the control animals. More interestingly, exosomes derived from the BALF in the septic rats had high levels of miR-92a-3p, which could be decreased by GW4869 pretreatment (**Figure 4F**). These results indicated that AEC-derived exosomes contain miR-92a-3p, which might serve as a pivotal miRNA participating in the pathogenesis of septic lung injury. Thus, we hypothesized that AEC-derived exosomes may transfer miR-92a-3p to alveolar macrophages, leading to the activation of macrophages during sepsis-induced ALI.

To explore the transmission of miR-92a-3p between AMs and AECs, we treated AECs with LPS. miR-92a-3p was enriched in secreted exosomes but not in cells. Alveolar macrophages treated with LPS-Exos exhibited higher expression of miR-92a-3p than untreated alveolar macrophages (**Figure 4G**). This finding indicates that LPS selectively enhances the loading of miR-92a-3p into exosomes from the cellular compartment and transfers them to recipient alveolar macrophages.

### AEC-Derived Exosomes Promote Alveolar Macrophage Activation *via* miR-92a-3p *In Vitro* and *In Vivo*

**Figure 5:**
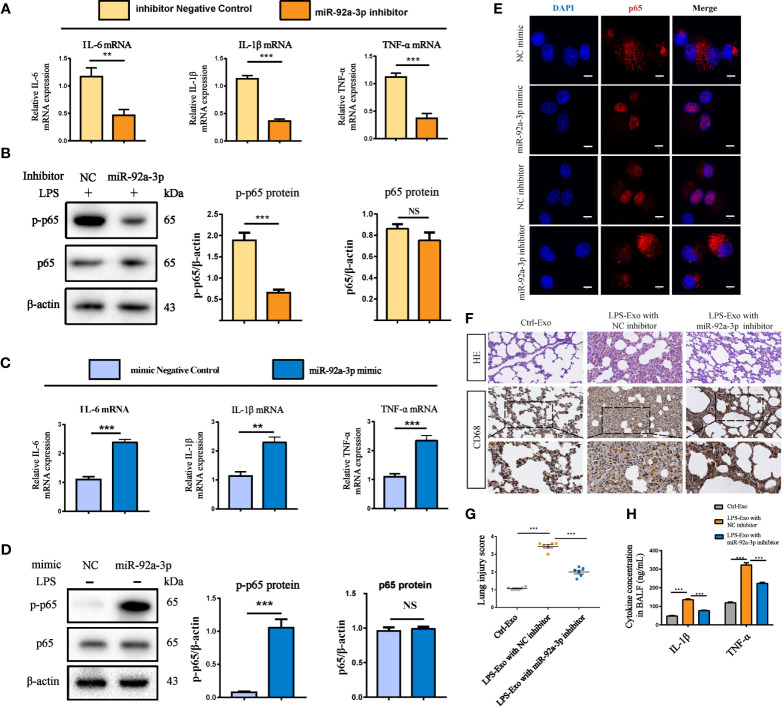
AEC exosome-derived miR-92a-3p promotes alveolar macrophage inflammation *in vitro* and *in vivo*. **(A, B)** Inflammatory cytokine expression and the NF-κB signalling pathway in macrophages treated with exosomes from LPS-induced AECs transfected with miR-92a-3p inhibitor or the corresponding NC. **(C, D)** Inflammatory cytokine expression and the NF-κB signalling pathway in macrophages treated with exosomes from AECs transfected with miR-92a-3p mimic or the corresponding NC. **(E)** Representative images of the nuclear translocation of p65, as observed by immunofluorescence assays, Scale bar, 5 μm. Data are presented as the mean ± S.E.M.**P<0.01, ***P<0.001 compared between two groups. NS, no significant difference, n=3 per group. For the *in vivo* experiment, AECs were cultured and treated with PBS or LPS for 24 h after transfection with NC or miR-92a-3p inhibitor. Exosomes were transferred to rats through intratracheal instillation. Rats were sacrificed 24 h after the treatment (n = 6 for each group) **(F)** Histologic (HE staining) changes and CD68 positive macrophage infiltration in the lung tissue. **(G)** The numbers of CD68 positive loci were quantified. **(H)** ELISAs showed the concentration of intrapulmonary inflammatory cytokines (IL-1β, TNF-α) in the BALF. Data are presented as the mean ± S.E.M. ***P<0.001 compared between two groups. n=6 per group.

To further confirm the roles of miR-92a-3p in alveolar macrophage activation during LPS-Exo exposure, we used a miR-92a-3p inhibitor, an anti-miR oligonucleotide, to inhibit the expression of miR-92a-3p. Inhibition of miR-92a-3p significantly reduced the levels of proinflammatory cytokines such as IL-1β, IL-6, and TNF-α and p65 phosphorylation in the LPS-Exo-treated recipient AMs (**Figures 5A, B**). In contrast, overexpression of miR-92a-3p with the miR-92a-3p mimic induced the expression of proinflammatory factors and activated the inflammatory signalling pathway in the Ctrl-Exo-treated recipient AMs (**Figures 5C, D**). Similar results were found for p65 translocation from the cytoplasm to the nucleus, indicating that AEC-exosomes promote alveolar macrophage activation through a miR-92a-3p dependent pathway (**Figure 5E** and **Figure S1** in the **Supplemental Material**).

To further investigate the roles of miR-92a-3p in AEC-derived exosomes *in vivo*, we isolated exosomes from the LPS-treated AECs in the presence of a miR-92a-3p inhibitor and transferred them to rats through airway instillation. Exosomes from the miR-92a-3p-inhibited AECs resulted in modest lung injury and decreased CD68+ AM infiltration while LPS-Exo treatment caused severe lung injury and intrapulmonary inflammation (**Figure 5F**). Accordingly, LPS-treated AEC-derived exosomes increased the expression of lung inflammatory cytokines compared to that of the control rats. AEC-derived exosomes with miR-92a-3p inhibition significantly decreased the levels of lung inflammation by downregulating the expression of the proinflammatory factors IL-1β and TNF-α (**Figures 5G, H**). The results suggest that AEC-derived exosomes encapsule miR-92a-3p, which can be delivered to the lung and trigger intrapulmonary inflammation.

### AEC Exosome-Encapsulated miR-92a-3p Activates NF-κB Signalling in Macrophages by Targeting PTEN

**Figure 6:**
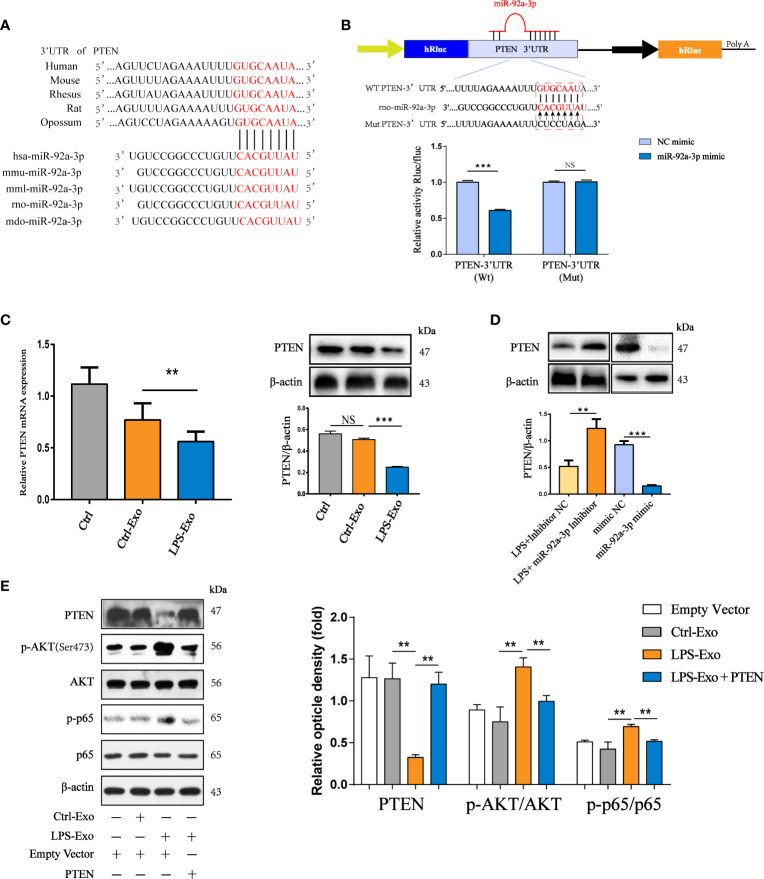
Exosomal miR-92a-3p activates alveolar macrophages *via* PTEN. **(A)** Conservation of the miR-92a-3p target sequence in the PTEN 3’UTR among different species and conservation of the miR-92a-3p sequence among different species. **(B)** The dual-luciferase reporter assay was performed in 293T cells. Cells were cotransfected with the wild-type or mutant PTEN 3’UTR luciferase reporter plasmids, as well as miR-92a-3p mimic or NC mimic. **(C)** mRNA and protein expression of PTEN in recipient alveolar macrophages treated with AEC-Exo were examined by RT-PCR and western blot analysis. **(D)** The protein levels of PTEN were detected using western blot analysis after transfection with miR-92a-3p inhibitor or miR-92a-3p mimic. β-actin was used as an internal control. **(E)** Immunoblot of lysates from AMs overexpressing PTEN in the presence or absence of LPS-Exos. The rescue of PTEN expression dramatically inhibited LPS-Exo induced p65 phosphorylation and tended to decrease these levels in response to LPS-Exos. β-actin was used as an internal control. Data are presented as the mean ± S.E.M. **P<0.01, ***P<0.001 compared between two groups. NS, no significant difference, n=3 per group.

To elucidate the mechanism by which LPS-Exo-derived miR-92a-3p induces AM activation, we used bioinformatics analysis to determine whether PTEN might be the putative target gene for miR-92a-3p, which is conserved across species (**Figure 6A**). Previous studies have revealed that PTEN acts as an inhibitor of the PI3K/AKT pathway, which has been associated with the regulation of NF-κB activation (Zhang et al., 2015). Therefore, a luciferase reporter assay was performed to assess the direct interaction between the 3′UTR of PTEN and miR-92a-3p. The results showed that the miR-92a-3p mimic significantly reduced the luciferase reporter activity of the WT-PTEN plasmid by approximately 40%, conversely, the activity of the PTEN 3’UTR–mutant luciferase reporter was not affected (**Figure 6B**). These data indicate that PTEN is a direct target of miR-92a-3p.

To examine whether AEC-derived exosomes induced by LPS transfer miR-92a-3p to regulate PTEN expression in recipient AMs, we treated AMs with LPS-Exos. As expected, both the mRNA and protein expression levels of PTEN were repressed by miR-92a-3p-enriched exosomes from the LPS-treated AECs in recipient AMs after 12 h of coculture (**Figure 6C**). The expression of PTEN was examined in AM treated with exosomes containing miR-92a-3p (**Figure 6D**). Exosomes from the miR-92a-3p mimic-transfected AECs strongly inhibited PTEN expression. However, the expression of PTEN was upregulated under inhibition of exosomal miR-92a-3p compared to administration of LPS-Exos. Thus, LPS-Exos shuttling of miR-92a-3p may activate NF-κB signalling in AMs by targeting PTEN. We examined whether the effects elicited by transferred exosomes could be reversed by increasing the PTEN levels. As shown in **Figure 6E**, increased levels of phosphorylated NF-κB were reduced upon PTEN overexpression in the AMs treated with LPS-Exos.

## Discussion

**Figure 7:**
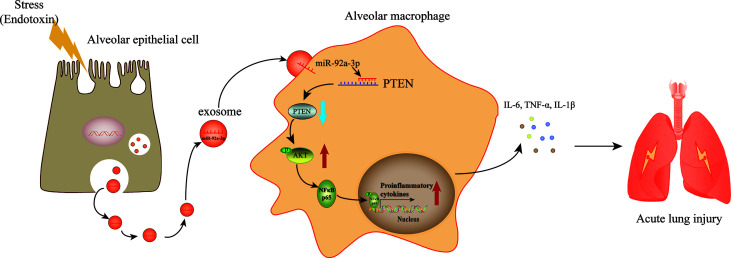
A schematic diagram showing the role of exosomal miR-92a-3p from LPS-induced AECs in inducing AM activation in septic lung injury. LPS stimulation induces AECs to release an increased number of exosomes, which are enriched with miR-92a-3p. The AEC-derived exosomes are taken up by AMs. miR-92a-3p induced degradation of the PTEN mRNA in AMs, resulting in the downregulation of the PTEN protein level and subsequent activation of the NF-κB signalling pathway. The nuclear translocation of p65 further promotes the release of proinflammatory factors in AMs.

The destruction of the epithelial barrier, pulmonary interstitial and alveolar spaces filled with protein-rich fluid and overwhelming inflammation are pathological features in ALI. However, the mechanism underlying damaged AEC-induced inflammation remains unclear. In the current study, we found that exosomes derived from LPS-treated AECs induced pulmonary inflammation and promoted alveolar macrophage activation (**Figure 7**). Our findings demonstrated a new mechanism by which AECs under septic conditions promote alveolar macrophage activation. Exosome-encapsulated miRNAs mediate cellular communication between AECs and AMs, which may provide a novel molecular target for the treatment of pulmonary inflammation.

Exosomes are small vesicles secreted by various types of cells. Exosomes are important mediators of cell-cell interactions and might participate in anti-inflammatory and proinflammatory effects during sepsis. In the present study, GW4869, an exosome release inhibitor, was applied to investigate the roles of exosomes in septic lung injury. GW4869 could attenuate the expression of proinflammatory cytokine levels during septic lung injury, which is consistent with the results of Okuro et al. (2018). In addition, our results showed that BALF from a septic rat model contained a number of exosomes, which could be reduced by GW4869 treatment. Pulmonary capillary permeability, lung water content and inflammatory cell infiltration in the lung were significantly alleviated after septic lung injury in rats treated with GW4869. GW4869 treatment also improved the survival rate of septic rats and decreased sepsis scores. This evidence suggests that exosomes derived from pulmonary cells may aggravate septic lung injury.

Increasing evidence has demonstrated that extracellular vesicles derived from epithelial cells induce intrapulmonary inflammation and contribute to the pathogenesis of sterile lung injury (Moon et al., 2015; Lee et al., 2016; Lee et al., 2017). Lee et al. (Lee et al., 2016; Lee et al., 2017) showed that microvesicles derived from alveolar epithelial cells stimulated by hyperoxia or acidic substances promote the migration and activation of macrophages. However, the roles and underlying mechanisms of alveolar epithelial cell-derived exosomes in septic lung injury remain to be determined. Given that we were unable to directly purify AEC-derived exosomes from the BALF of a septic rat model, we used LPS to stimulate alveolar epithelial cells to mimic inflammatory pathogenesis *in vitro*. The content of AEC-derived exosomes was robustly increased in BALF from the septic rat model. In addition, we found that the number of exosomes was elevated after LPS treatment of AECs *in vitro*. We conclude that AECs release more exosomes than normal AECs under exposure to LPS or a septic environment.

Alveolar macrophages are key regulators initiating the inflammatory response in sepsis-induced lung injury. Through tracking exosomes with PKH-67, we observed that LPS-treated AEC exosomes were internalized by AM *in vitro* and *in vivo*. This process leads to the activation of the NF-κB signalling pathway and the expression of proinflammatory cytokines in AMs, which contributes to the pathogenesis of acute lung injury.

miRNAs are the main components that shuttle in exosomes and regulate the biological functions of recipient cells by inducing translational repression and mRNA degradation. Under various pathological conditions, specific miRNAs might be transferred to exosomes to play functional roles (He et al., 2018). Our results showed that exosomes from LPS-treated AECs had significantly different expression profiles of miRNAs compared to normal controls. We identified 26 differentially expressed miRNAs in the LPS-treated AEC exosomes. In particular, miR-92a-3p was the most enriched miRNA in the LPS-treated AECs. Exosomes isolated from BALF in CLP-induced septic lung injury *in vivo* and cultured AECs *in vitro* had high levels of miR-92a-3p compared to those of the controls. To our surprise, the expression of miR-92a-3p was decreased in alveolar epithelial cells after LPS stimulation. In this regard, we believe that LPS stimulation promotes selective loading of miR-92a-3p into extracellular vesicles and reduces intracellular levels. miR-92a-3p has been reported to be a biomarker for the diagnosis and prognosis of sepsis-induced ARDS (Xu et al., 2020). Inhibition of miR-92a-3p reduced the pathological progression of ischemia-reperfusion-induced inflammation in large animal models (Hinkel et al., 2013). Our data showed that overexpression of miR-92a-3p by mimics enhances inflammatory responses in alveolar macrophages. In contrast, miR-92a-3p-depleted exosomes from LPS-treated AECs reversed the inflammatory effects. All these results suggest that miR-92a-3p is associated with the inflammatory responses. miR-92a-3p encapsulated in AEC exosomes may have biological functions in regulating septic lung injury.

The exact mechanism by which miR-92a-3p causes macrophage activation requires further investigation. The possible mRNA targets of miR-92a-3p were predicted by TargetScan assays. Among all candidate mRNAs, PTEN has been studied in the context of acute lung injury inflammation (Rao et al., 2015; Zhou et al., 2019). A dual-luciferase assay was performed to confirm that miR-92a-3p can bind to the predicted binding site in the PTEN 3’UTR. The data here showed a significant decrease in PTEN protein and mRNA expression in AMs after treatment with AEC-derived exosomes enriched with miR-92a-3p. Recently, studies have found that PTEN can negatively regulate the PI3K/AKT pathway which is an upstream activator of the NF-κB signalling cascade and exerts an anti-inflammatory effect (Feng et al., 2014; Chen et al., 2020). The results potently showed that LPS-treated AEC exosomes induce alveolar macrophage inflammation through inhibition of PTEN expression.

In conclusion, we demonstrated that exosomes derived from LPS-treated AECs induce pulmonary inflammation. Exosome-shuttled miR-92a-3p mediated the crosstalk between AECs and AMs, which contributes to macrophage activation by inhibiting PTEN expression and regulating the activation of the NF-κB signalling pathway. Further, our findings suggest that miR-92a-3p in AEC-derived exosomes might represent a novel diagnostic biomarker for septic lung injury. Treatment targeting miR-92a-3p may be a new therapeutic strategy for alleviation of septic lung injury.

## Data Availability Statement

The original contributions presented in the study are included in the article/**Supplementary Material**. Further inquiries can be directed to the corresponding author.

## Ethics Statement

Animal experimental procedures and protocols were approved by the ethics committee of the Animal Experimental Research Center of Nanchang University (No. NCDXSYDWLL-2017619).

## Author Contributions 

KQ conceived ideas and contributed to project planning. FL improved the quality of the manuscript and performed most of the experiments. WP wrote the paper and analyzed the data. JC and ZX performed the rats experiments. RJ, QS and NZ conceived the idea for the workshop. All authors contributed to the article and approved the submitted version.

## Funding

This research was funded by National Natural Science Foundation of China (81671894 and 81660315) and the Natural Science Foundation of Jiangxi Province (20202BABL206069).

## Conflict of Interest

The authors declare that the research was conducted in the absence of any commercial or financial relationships that could be construed as a potential conflict of interest.

## Abbreviations

ALI, Acute Lung Injury; AM, Alveolar Macrophage; AEC, Alveolar Epithelial Cell; BALF, Bronchoalveolar lavage fluid; Ctrl, Control; Exos, Exosomes; NC, Negative control; NF-κB, nuclear transcription factor-κB; NS, Not significant; PBS, Phosphate buffer saline; PTEN, Phosphate and tension homology deleted on chromsome ten; p-p65, Phospho- nuclear transcription factor-κB.
