# Supplementary material for: Exosomes Derived From Alveolar Epithelial Cells Promote Alveolar Macrophage Activation Mediated by miR-92a-3p in Sepsis-Induced Acute Lung Injury
Source: Front Cell Infect Microbiol. 2021 May 10;11:646546. doi: 10.3389/fcimb.2021.646546 (PMC8141563; doi:10.3389/fcimb.2021.646546)
Supplement: Supplementary file 2 [file DataSheet_1.docx]

**Figure S1**


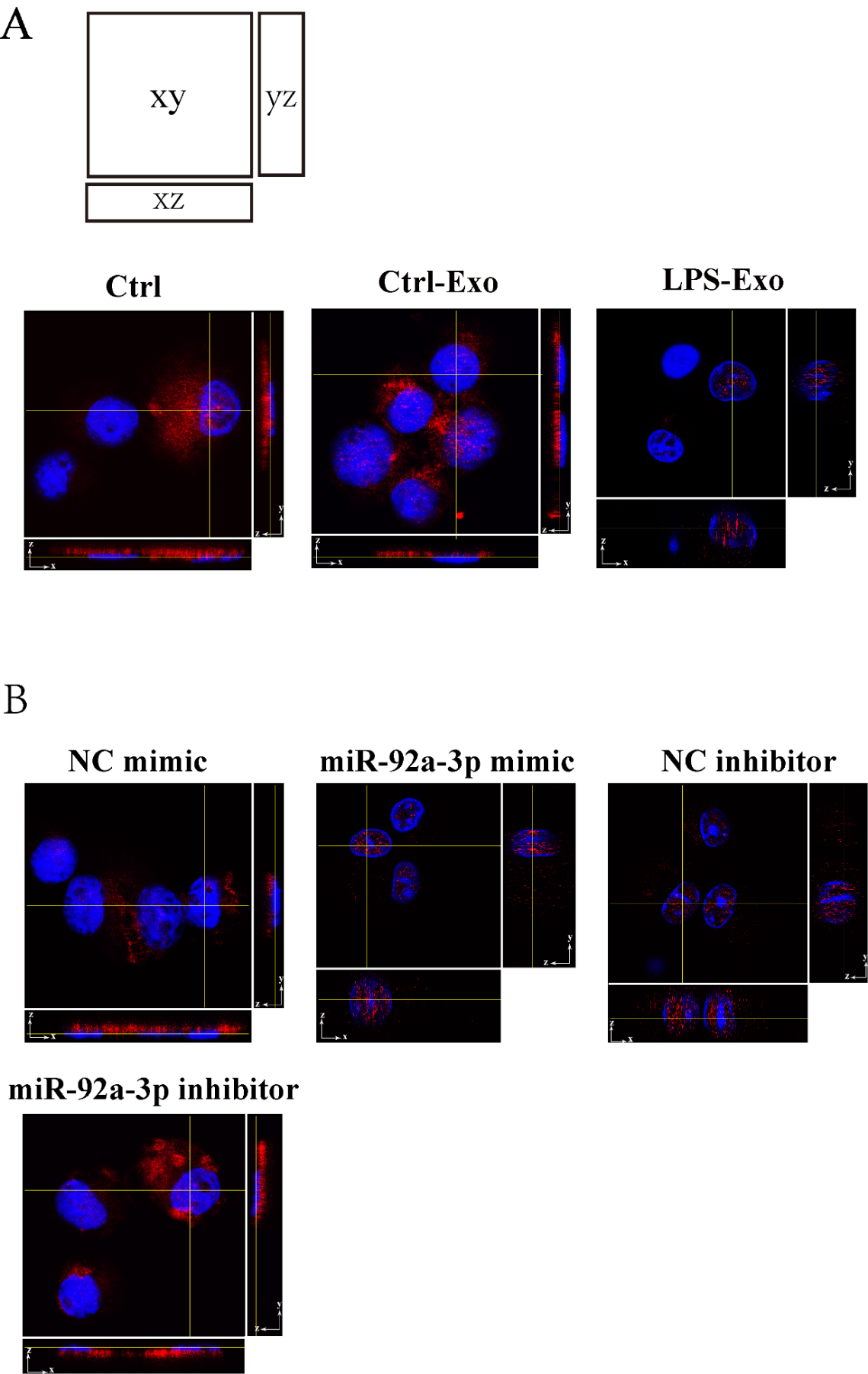


Three-dimensional image analysis showing the nuclear translocation of Nuclear factor-kB (NF-kB) p65 in AM. Images from confocal microscopy with z-stacking were analyzed by XY, XZ, and YZ sections.
